# Knowledge, Attitude, and Practice of Standard Infection Control Precautions Among Medical Students at King Khalid University Hospital

**DOI:** 10.7759/cureus.62768

**Published:** 2024-06-20

**Authors:** Sulaiman A Alshammari, Shahad S Alrasheed, Wojoud A Alruhaimi, Aljoharah I Albnyan, Batoul Alruhaimi, Maryam Hajj

**Affiliations:** 1 Family and Community Medicine, College of Medicine, King Saud University, Riyadh, SAU; 2 Family and Community Medicine, King Saud University Medical City, College of Medicine, King Saud University, Riyadh, SAU; 3 Medicine, King Saud University, Riyadh, SAU; 4 Infectious Diseases, King Saud University Medical City, College of Medicine, King Saud University, Riyadh, SAU

**Keywords:** knowledge, medical students, attitude and practices, infection control, standard precautions

## Abstract

Background

Healthcare-associated infection (HAI) risk extends beyond patients to healthcare workers and medical students. However, many HAIs are preventable by adhering to standard infection control precautions (SICPs). This study assesses medical students’ knowledge, attitudes, and practices regarding SICPs at the College of Medicine, King Saud University.

Methodology

A cross-sectional study was undertaken at the College of Medicine, King Saud University, Riyadh, Saudi Arabia, involving interns and medical students from years 1 to 5. The researchers constructed and validated an electronic questionnaire, which was used for data collection from December 2022 to June 2023. The estimated sample size was 371 students and interns, stratified by sex and educational level. The data collected included demographic characteristics, knowledge, attitudes, and practices regarding SICPs.

Results

Out of 371 participants, approximately a quarter (25.1%) had a good knowledge score, 25.6% had a positive attitude, and 26.7% had good infection control practice. There was an association between good knowledge and male gender; being in clinical years 3, 4, 5, and interns; and attending infection control training (p < 0.05). A significant difference in practice was noted among females (p = 0.002).

Conclusions

This study showed low-to-moderate knowledge, attitude, and practice toward SICPs. These findings indicate a gap between the recommended guidelines and their implementation in healthcare settings, highlighting the necessity for integrating infection control education throughout the medical curriculum. Further research involving a larger sample from multiple institutions is warranted.

## Introduction

Nosocomial infections, i.e., healthcare-associated infections (HAIs), first appear 48 hours after hospitalization or within 30 days after receiving health care [1]. HAIs significantly contribute to healthcare expenditures in developed and developing nations by causing mortality, morbidity, prolonged hospital stays, economic strain on public health, and the advent of multidrug-resistant strains of microorganisms [2]. The universal rate of HAIs is 0.14%, with an annual increase of 0.06%, and the highest rates are reported in Africa and Asia [1]. Patients admitted to acute care health facilities are in danger of contracting such HAIs, as 5-10% of them acquire an infection. Moreover, the risk of HAIs extends beyond patients to include healthcare workers (HCWs) and medical students, particularly if there is poor adherence to standard infection control precautions (SICPs) [3]. However, many HAIs are preventable by following SICPs, which refer to HCW practices to reduce infection transmission, including hand hygiene, personal protective equipment (PPE), respiratory hygiene, and safe injection practices [4]. Compliance with SICPs is an effective and inexpensive measure that helps reduce the transmission of infectious diseases and HAIs, directly proportional to patient cost and hospital stay [5,6]. For instance, it has been estimated that the annual costs of HAIs in the United States range from 7.2 to 14.9 billion dollars [7]. However, several factors have been found to influence compliance with SICPs. For example, it has been reported that lack of education, heavy workload, equipment unavailability, and resources influence nurses’ adherence to SICPs [5]. Studies evaluating medical students’ knowledge and attitudes toward infection control measures concluded that they needed more training. Standard precautions are widely promoted, and many guidelines are issued globally in hospital settings [2,8,9]. However, according to one study, only 13% of students reviewed infection control guidelines before clinical training [10]. Poor knowledge was found in hand hygiene, PPE, and sharps control, as half of the students with needle-stick injuries should have reported it [9,11]. However, a study conducted in Jordan found that medical students needed more knowledge and attitudes about infection prevention and control measures. Good knowledge and attitudes were found in cough etiquette, respiratory hygiene, hand hygiene, and infection transmission through hospital attire and equipment [3]. Studies concluded that acquiring good knowledge, a positive attitude, and practice of SICPs is essential to prevent the transmission of infections [12-14]. Our literature search showed a few studies assessing the knowledge, attitudes, and practices (KAP) of SICPs in Saudi Arabia. These studies investigated KAP among the public [15] and in healthcare settings. Most studies conducted locally involved the dental field [16-18]. One study evaluated knowledge among health science students [19], two studied infection control among HCWs in Qassim and Abha [12,20], two studies focused on hand hygiene among health science students and HCWs [13,21], and one assessed prevention and control of nosocomial infections among HCWs and non-HCWs [22]. A study conducted a decade ago at King Faisal University in Al-Ahsa, Saudi Arabia, investigated medical students’ knowledge.

Nevertheless, the practical aspects of infection control procedures still need to be evaluated. The investigators concluded that health personnel in hospitals and primary care settings must learn and adhere to such protocols [9]. In the last 10 years, the world suffered several pandemics, including COVID-19. There needs to be more data about the knowledge and attitudes of medical students regarding SICPs. Therefore, this study aims to assess the KAP among medical students in the College of Medicine, King Saud University, Saudi Arabia. Furthermore, the study aims to investigate students’ views toward the existing curriculum in providing them with effective knowledge and essential skills related to SICPs.

## Materials and methods

Study design

A cross-sectional study was conducted among medical students and interns at the College of Medicine, King Saud University, Riyadh, Saudi Arabia, from December 2022 to June 2023. We used stratified sampling techniques based on sex and educational level. The minimum sample size of participants was calculated as follows [23]: n = Z^2^ × P(1-P)/d^2^, where n is the calculated sample size, Zα is 1.96 for 95% confidence level, P is the proportion of those with good knowledge = 0.267 (based on a previous study) [9], and D is the maximum acceptable error = 0.05. The calculated minimum sample size (n) was 300. We added 20% to account for non-response and incomplete responses, making the total sample size 360.

Questionnaire

The questionnaire was designed based on three similar previous studies [3,12,20], which was reviewed by an infection control consultant. The questionnaire was divided into four sections. The initial section comprised inquiries about demographics, whereas the subsequent three sections assessed knowledge, attitude, and implementation of SICPs. To measure knowledge, we used 31 questions. We awarded 1 point for every correct answer and 0 for every incorrect answer, with knowledge scores ranging from 0 to 31. The respondents’ attitudes were assessed using a Likert scale. The participants’ attitudes were reported as strongly agree, agree, neutral, disagree, and strongly disagree. A score of 1 was given for agreeing with positive attitude questions or disagreeing with negative attitude questions. The attitude score ranged from 0 to 9. A set of 12 practice questions measured the practice score, with some questions measured by Likert-scale responses (always, most of the time, sometimes, rarely, and never). A score of 1 was given for every correct practice and 0 for incorrect ones, with the score ranging from 0 to 12. The participants whose total KAP scores were in the 75th percentile or higher were considered to have good KAP. Scores below the 75th percentile were ranked as poor-to-moderate KAP.

Data collection method

The questionnaire was sent to students online via e-mail and WhatsApp using Google Forms. A total of 10 male and 10 female students from the medical college at King Saud University participated in a pilot study to ensure clarity, comprehensibility, and the estimated time to complete the questionnaire. The participants spent 7-10 minutes completing the questionnaire. Inclusion criteria included medical students (from years 1 to 5) and interns at the College of Medicine, King Saud University.

Ethical considerations

The King Saud University, College of Medicine Institutional Review Board approved the study on December 25, 2022 (project number E-22-7413). The online survey included a short introduction message describing the study’s aim. All respondents signed a written consent form before completing the electronic questionnaire. The questionnaire was anonymously obtained to ensure confidentiality, and participants had the right to withdraw from the study at any time.

Statistical analysis

Data were analyzed using SPSS Statistics for Windows, version 26 (IBM Corp., Armonk, NY, USA). Frequencies and percentages were reported for categorical variables and were compared using the chi-square test. Continuous variables were tested for distribution using the Shapiro-Wilk test and were reported as median with interquartile range (IQR). Adjusted odds ratios (AORs) and corresponding 95% confidence intervals (CIs) were utilized to present the findings. A p-value <0.05 was considered statistically significant [23,24].

## Results

The study included 371 medical students and interns. Males and females were distributed evenly. Their age ranged from 18 to 27 years, most in the age groups of 20-21 years (30.7%) and 22-23 years (37.2%). The five academic years and interns were almost equally represented. Most participants (99.2%) had received the COVID-19 vaccine, while only 68.4% and 44.7% had received hepatitis B and influenza vaccines, respectively. Most participants (93.8%) had no medical conditions. Fewer than half of the participants (42.9%) had attended infection control training programs (Table 1).

**Table 1:** Characteristics of the study participants (N = 371).

Characteristics		N	%
Gender	Female	186	50.1%
	Male	185	49.9%
Age groups, year	18–19	40	10.8%
	20–21	114	30.7%
	22–23	138	37.2%
	24–25	68	18.3%
	26–27	11	3.0%
Academic year	First year	61	16.4%
	Second year	64	17.3%
	Third year	63	17.0%
	Fourth year	60	16.2%
	Fifth year	62	16.7%
	Intern	61	16.4%
Received vaccinations	COVID-19 vaccine	368	99.2%
	Hepatitis B vaccine	253	68.4%
	Influenza vaccine	166	44.7%
	Meningococcal vaccine	80	21.6%
	Pneumococcal vaccine	70	18.9%
History of medical diseases	No	348	93.8%
	Bronchial asthma	20	5.4%
	Hypertension	1	0.3%
	Prediabetes	1	0.3%
	Ulcerative colitis	1	0.3%
Attended a training program in infection control or standard precautions		159	42.9%

Knowledge

**Figure 1:**
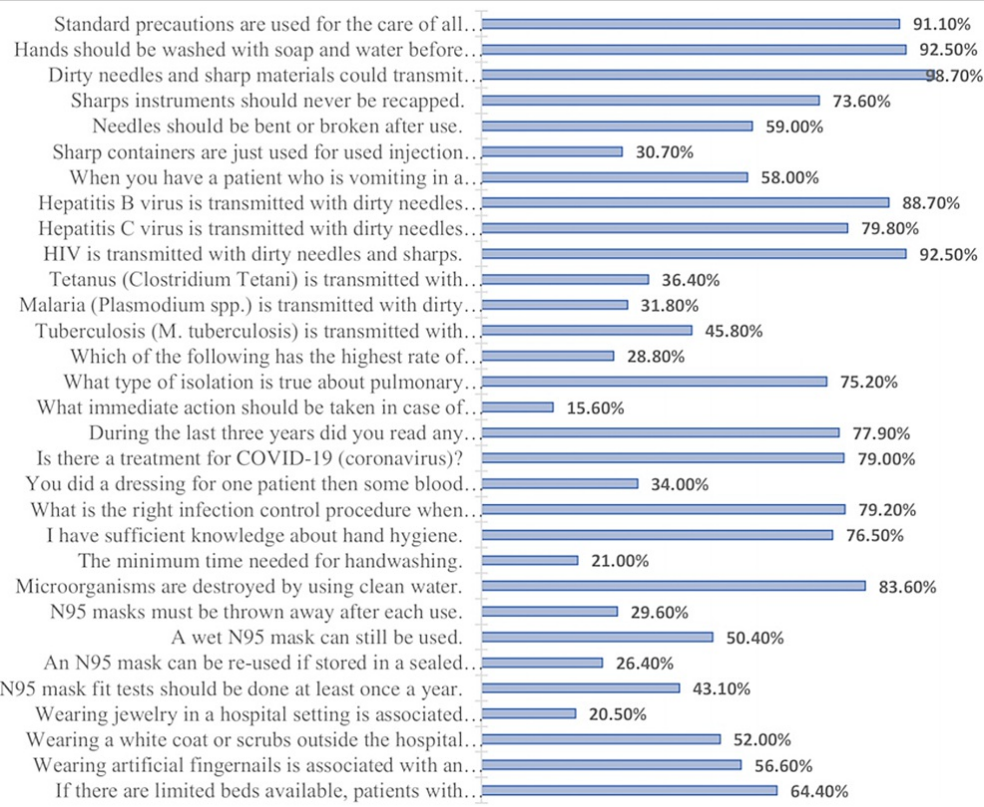
Correct responses regarding knowledge of infection control measures (N = 371).

Table 2 and Figure 1 show correct responses regarding knowledge of infection control measures. The most correctly answered statements were that contaminated needles and sharp materials could transmit disease-causing agents (98.7%), standard precautions are utilized for all patient care (91.1%), and that even with gloves, hands should always be cleansed with water and soap before and after handling potentially infectious objects (92.5%).

**Table 2:** Correct responses regarding knowledge of infection control measures (N = 371). WHO = World Health Organization; MOH = Ministry of Health; CDC = Centers for Disease Control and Prevention; HIV = human immunodeficiency virus

Correct responses regarding knowledge of infection control measures	N (%)
Standard precautions are used for the care of all patients regardless of their diagnosis and perceived infection status	338 (91.1)
Hands should be washed with soap and water before and after handling potentially infectious materials irrespective of wearing gloves	343 (92.5)
Dirty needles and sharp materials could transmit disease-causing agents	366 (98.7)
Sharps instruments should never be recapped	273 (73.6)
Needles should be bent or broken after use	219 (59.0)
Sharp containers are just used for used injection needles	114 (30.7)
When you have a patient who is vomiting in a dressing room or clinic first step infection control procedure is to isolate the infected area	215 (58.0)
Hepatitis B virus is transmitted with dirty needles and sharps	329 (88.7)
Hepatitis C virus is transmitted with dirty needles and sharps	296 (79.8)
HIV is transmitted with dirty needles and sharps	343 (92.5)
Tetanus (*Clostridium tetani*) is transmitted with dirty needles and sharps	135 (36.4)
Malaria (*Plasmodium* spp.) is transmitted with dirty needles and sharps	118 (31.8)
Tuberculosis (*Mycobacterium tuberculosis*) is transmitted with dirty needles and sharps	170 (45.8)
Which of the following has the highest rate of transmission via saliva?	107 (28.8)
What type of isolation is true about pulmonary tuberculosis?	279 (75.2)
What immediate action should be taken in case of direct blood contact with an HIV patient?	58 (15.6)
During the last three years did you read any information about COVID-19 from verified sources such as WHO, MOH, or CDC?	289 (77.9)
Is there a treatment for COVID-19?	293 (79.0)
You did a dressing for one patient and some blood coming from the wound contaminated your exposed skin, the best disinfection material to clean your skin is	126 (34.0)
What is the right infection control procedure when the finger is pricked by an intravenous line needle?	294 (79.2)
I have sufficient knowledge about hand hygiene	284 (76.5)
The minimum time needed for handwashing	78 (21.0)
Microorganisms are destroyed by using clean water	310 (83.6)
N95 masks must be thrown away after each use	110 (29.6)
A wet N95 mask can still be used	187 (50.4)
An N95 mask can be reused if stored in a sealed plastic bag	98 (26.4)
N95 mask fit tests should be done at least once a year	160 (43.1)
Wearing jewelry in a hospital setting is associated with an increased risk for the spread of diseases	76 (20.5)
Wearing a white coat or scrubs outside the hospital setting is associated with an increased risk for the spread of diseases	193 (52.0)
Wearing artificial fingernails is associated with an increased risk for the spread of diseases	210 (56.6)
If there are limited beds available, patients with communicable diseases may be admitted to the same ward mixed with other patients	239 (64.4)

Moreover, contaminated needles and sharp materials could transmit disease-causing agents (98.7%). Knowledge about the disposal of sharp instruments was variable; 73.6% knew that sharp instruments should never be recapped, while 59.0% knew that needles should not be bent or broken after use, and only 30.7% recognized that sharp containers are just used for used injection needles. Most participants (79.2%) identified that the right action after pricking the finger with a needle is dressing the wound and informing the infection control practitioner. High percentages knew that human immunodeficiency virus (HIV) (92.5%), hepatitis B virus (88.7%), and hepatitis C virus (79.8%) are transmitted by contaminated needles and sharp instruments. In contrast, their knowledge about transmission of tetanus (36.4%), malaria (1.8%), and tuberculosis (45.8%) was poor. More than half (58%) of the respondents correctly identified isolation of the infected area as the initial action when confronted with a vomiting patient. Knowledge about taking anti-HIV drugs immediately after direct blood contact with an HIV-positive person was very low (15.6%). Approximately two-thirds (64.4%) of the respondents correctly stated that patients with infectious diseases should not be admitted to the same ward as other patients when bed availability is limited. Approximately one-third of the participants (34%) identified soap and water as the most effective disinfectant for cleansing exposed skin following contamination. The level of knowledge regarding the bare minimum time needed for handwashing was relatively low (21.0%). Low levels of knowledge were found regarding the following precautions associated with the use of N95 masks: not reusing wet N95 masks (50.4%), conducting N95 mask fit tests annually (43.1%), not discarding N95 masks after each use (29.6%), and the possibility of reusing N95 masks stored in a sealed plastic bag (26.4%). In addition, only 20.5% strongly agreed that wearing jewelry in a hospital setting increases the risk of spreading disease. In comparison, 52% and 56% strongly agreed that wearing a white coat or scrubs, respectively, outside the hospital increases the risk of spreading diseases.

Attitudes

Table 3 shows the participants’ positive attitudes toward infection control measures. The highest positive attitudes were observed for preferring hand hygiene before and after each interaction with patients (77.9%) and following strict infection control practices when dealing with tuberculosis patients (74.4%). However, attitudes were less positive toward the protective role of standard precautions against acquiring infections from the healthcare facility (43.4%) and the importance of using gloves for all patient care to reduce the risk of microbial transmission (44.7%). In addition, only 19.1% strongly agreed that there is a high risk of occupational infection among HCWs.

**Table 3:** Positive attitudes toward infection control measures (N = 371).

Participants’ positive attitudes toward infection control measures	N	%
Standard precautions will prevent acquiring infections from the healthcare facility	161	43.4%
There is no need to wash or decontaminate hands after touching patients’ surroundings	241	65.0%
Using gloves with all patient care is a useful strategy for reducing the risk of transmission of microbes	166	44.7%
In the absence of standard precautions, healthcare facilities can be the source of infection and epidemic diseases	261	70.4%
The risk of occupational infection among health workers in my workplace is high	71	19.1%
Strict infection control practices should be followed when dealing with tuberculosis patients	276	74.4%
Prefers to perform hand hygiene before and after any interaction with patients	289	77.9%
Transmission of infectious organisms can be reduced by adhering to standard and contact precautions	237	63.9%
It is not logical to assume all patients are contagious unless their infection has been confirmed	75	20.2%

Practices

Table 4 shows the correct infection control practices of the study participants. Approximately 64% reported always washing their hands before and after each patient encounter, while 48.0% always used gloves when examining all patients. In addition, 72.0% always used a face mask when examining all patients with suspected infections, while 35% always wore goggles/face masks during procedures. The frequency of wearing a medical gown when dealing with infectious patients was 53.4%. Respiratory hygiene and etiquette were maintained when coughing/sneezing using a disposable napkin and washing hands (91.1%) or over the shoulder if a napkin is unavailable (76.5%).

**Table 4:** Correct infection control practices among the study participants (N = 371).

Correct infection control practices of the study participants	N	%
To what extent do you wash your hands before examining all patients and after each patient encounter?	238	64.2%
To what extent do you recap needles immediately after using them?	133	35.8%
To what extent do you use gloves while examining all patients?	178	48.0%
To what extent do you use a face mask while examining all patients with suspicious infections?	267	72.0%
To what extent do you wear goggles/face masks during procedures?	130	35.0%
To what extent do you wear a medical gown during interaction with infectious patients?	198	53.4%
Ever had a contaminated needle-stick injury?	341	91.9%
I do not have to wash my hands if I use gloves	330	88.9%
You can handle body fluids with bare hands if gloves are not available	284	76.5%
Which of the following sentences is true regarding respiratory hygiene and cough etiquette?		
Cough/sneeze on a disposable napkin and wash your hands	338	91.1%
Cough/sneeze over the shoulder if a napkin is not available	284	76.5%
Wipe your hands on the inside of your white coat after coughing or sneezing	271	73.0%

The participants’ median total knowledge score was 18 (IQR = 16-21), with their scores ranging from 2 to 28. The attitude score ranged from 0 to 9 with a median of 5 (IQR = 3-7). The practice score ranged from 1 to 10 with a median of 7 (IQR = 6-9). Approximately a quarter (25.1%) had a good knowledge score, 25.6% had a positive attitude, and 26.7% had good infection control practice.

Associations between basic characteristics of the study participants and KAP levels of infection control measures

Table 5 shows the significant associations between good knowledge and young age (18 to <24), male sex, being in the third, fourth, and fifth year of being an intern, and having attended an infection control training program (all p-values <0.05). Regarding attitudes, there was a significant association between being in the second year and having a positive attitude (p = 0.003). Furthermore, better practices were utilized by females at a significantly higher rate than by males (p = 0.002). Attending a training program was also significantly associated with good practice scores (p = 0.006).

**Table 5:** Associations between the characteristics of the participants and the levels of knowledge, attitude, and practices of infection control measures.

Participant characteristics		Knowledge score				Attitude score				Practices score			
		Poor/Moderate, N = 278 (74.9%)		Good, N = 93 (25.1%)		Poor/Moderate, N = 276 (74.4%)		Positive, N = 95 (25.6%)		Poor/Moderate, N = 272 (73.3%)		Good, N = 99 (26.7%)	
Age, years, N, %	18 to <24	235	84.5	57	61.3	218	79.0	74	77.9	219	80.5	73	73.7
	24 to 27	43	15.5	36	38.7	58	21.0	21	22.1	53	19.5	26	26.3
	P-value	0.001				0.823				0.158			
Sex, N, %	Female	150	54	36	38.7	142	51.4	44	46.3	123	45.2	63	63.6
	Male	128	46	57	61.3	134	48.6	51	53.7	149	54.8	36	36.4
	P-value	0.011				0.388				0.002			
Academic, year, N, %	First	58	20.9	3	3.2	50	18.1	11	11.6	45	16.5	16	16.2
	Second	56	20.1	8	8.6	36	13	28	29.5	47	17.3	17	17.2
	Third	54	19.4	9	9.7	54	19.6	9	9.5	52	19.1	11	11.1
	Fourth	46	16.5	14	15.1	44	15.9	16	16.8	39	14.3	21	21.2
	Fifth	35	12.6	27	29	48	17.4	14	14.7	49	18	13	13.1
	Intern	29	10.4	32	34.4	44	15.9	17	17.9	40	14.7	21	21.2
	P-value	<0.001				0.003				0.196			
Attended training program, N, %	No	187	67.3	25	26.9	156	56.5	56	58.9	167	61.4	45	45.5
	Yes	91	32.7	68	73.1	120	43.5	39	41.1	105	38.6	54	54.5
	P-value	<0.001				0.68				0.006			

## Discussion

The study’s findings suggest that only around one-fourth of medical students had good KAP. This finding was consistent with a study conducted among medical students at King Faisal University in 2013 (26.7%) [9]. However, the results contrasted with a survey in 2018, which found that most King Saud Bin Abdulaziz University for Health Sciences students demonstrated sufficient knowledge (73.6%) [19]. This variation can be explained by the low implementation of infection control training courses, as only 42.9% of this study’s participants attended training programs for standard precautions. Therefore, knowledge about infection control and standard precautions needs improvement and emphasis in the curriculum of preclinical and clinical years through frequent education and assessment [2].

Knowledge

Despite having low knowledge, most participants answered correctly to the knowledge statement related to general concepts of standard precautions and hand hygiene. These results were consistent with studies conducted in Jordan and Saudi Arabia [3,19]. However, only 21% identified that the minimum time required for handwashing is 20 seconds, and 76.5% stated having sufficient knowledge about hand hygiene, which is lower compared to findings among medical students in Jordan (92.7%) and Qatar (85.48%) in 2021 and 2016, respectively [2,3]. Authorities made a huge effort to promote preventive measures and mitigate the spread of COVID-19 infection among the Saudi public. These actions included the lockdown of all educational and recreational activities and mass gatherings, launching online learning [25], and virtual clinical consultation [26]. In addition, several social media applications were made available to the public to raise people’s perception of susceptibility and awareness of the seriousness of COVID-19 and promote preventive behavior [27]. The persistent use of PPE, such as facial masks, gloves, and, for frontline workers, shields and protective garments, is necessary. Family physicians and teams should play a vital role in this battle [28]. Medical students and trainees, in general, are exposed to infectious diseases through contact with patients and potentially contaminated equipment in the hospital. Thus, adequate knowledge about such diseases is crucial to prevent the spread of infections [2]. The majority of participants were aware that the hepatitis B virus, hepatitis C virus, and HIV are transmitted through the use of dirty needles. However, fewer than half of misidentified malaria and tuberculosis as being transmitted by dirty needles and sharps. Moreover, only 28.8% identified that tuberculosis has the highest transmission rate via saliva.

Additionally, knowledge about the correct action of administering anti-HIV drugs immediately in case of direct blood contact with HIV-positive patients was considerably low (15.6%); similar results were found among medical students in Jordan [3]. The extensive campaign to promote vaccination succeeded in increasing the level of vaccine intake to COVID-19 vaccine (99.2%), hepatitis B vaccine (68.4%), and Influenza vaccine (44.7%)compared to the early phase of the pandemic [29]. In addition, most participants (73.6%) knew that sharp instruments should never be recapped, which is higher compared to findings among medical students at King Faisal University (17.9%) and students at King Saud Bin Abdulaziz University for Health Sciences (36.45%) [9,19]. More than half of the students (59%) knew needles should not be bent or broken after use. However, only 30.7% recognized that sharp containers are just used for used injection needles. Most participants identified the right action after pricking the finger with a needle as dressing the wound and informing the infection control practitioner, contrary to a study conducted in Pakistan in 2018, which showed that medical students have low knowledge regarding needle-stick injuries, in which they encouraged students on reporting needle-stick injuries and familiarizing them with guidelines required in the hospital setting [11]. The findings of this study revealed that 20.5% of students responded affirmatively to statements assessing the potential for disease transmission via hospital attire and accessories. On the other hand, previous investigations documented proportions of 13.46% and 44.10% among medical students. In addition, our study showed that 52% of students concurred that they were aware of the disease transmission consequences of wearing white coats or scrubs outside of a hospital environment.

In contrast, the corresponding percentages in the studies mentioned above were 54.90% and 31.90% [2,3]. Furthermore, about 56.6% of medical students concurred that using artificial nails could facilitate disease transmission, whereas the percentages of agreement in the compared studies were comparatively lower at 33.90% and 45.10% [2,3]. Furthermore, a previous survey of health workers found that despite their favorable attitude toward surgical masks, they possessed low levels of knowledge and good practice [30].

Attitudes

The current findings demonstrated that participants held favorable attitudes toward SICPs, particularly toward hand hygiene before and after every patient interaction, with 77.9% expressing a preference for this precautionary measure to mitigate the potential for infection. This finding was comparable to HCWs in Al-Qassim in 2021 [12] and medical students in Jordan and Namibia [3,8]. In addition, numerous studies [10,13,31] conducted among medical students in Saudi Arabia revealed generally positive attitudes toward hand hygiene. According to these studies, a greater understanding of hand hygiene was significantly correlated with favorable attitudes [13,31]. Furthermore, a considerable percentage (74.4%) exhibited favorable dispositions toward adhering to stringent infection control protocols when interacting with tuberculosis patients. This result was consistent with the findings of a Jordanian study [3], which found that two-thirds of students exhibited this positive attitude when interacting with patients patients. The findings suggest that these particular infection control measures are well-received, implying that their significance in preventing the transmission of infections is acknowledged. Similar to a study conducted in Abha in 2021 [20], attitudes were less favorable regarding the protective function of standard precautions against contracting infections from the healthcare facility and the significance of wearing gloves during all patient care to reduce the risk of microbial transmission.

Moreover, approximately 19.1% of respondents agreed that healthcare personnel are exposed to a high risk of occupational infections on the job. The issue may arise from the restricted practical experience medical students and interns may have throughout their training, particularly in the initial phases of their academic journey. Consequently, their understanding of the documented occupational hazards and infections within healthcare environments may need to be improved.

Furthermore, this may be accounted for by the students’ trust or overconfidence in their training environment. Adherence to infection control protocols by medical students and interns is essential in ensuring patient safety and healthcare quality. According to this research, a mere 25% of medical students adhered to effective infection control protocols. This finding is similar to previous regional studies that observed inadequate adherence to SICPs [20]. Moreover, these findings may indicate that experience could influence the implementation and observance of SICPs [12,21].

Practices

Regarding practices among the participants, most students reported always washing their hands before examining patients, and fewer than half always used gloves during patient examinations. These results are concerning, given that proper glove use and hand hygiene are fundamental components of SICPs [4,5]. Hand hygiene is a cornerstone of infection control and should be practiced irrespective of glove use, as gloves do not provide complete protection against hand contamination [4]. The lack of consistent glove use may be attributed to a lack of resources or awareness, highlighting a critical improvement area in infection control practices [10]. Interestingly, nearly 72% of participants wore face masks when treating patients suspected of infections. This practice may reflect increased awareness and urgency of respiratory precautions, especially after recent pandemics such as COVID-19 [14]. However, the use of other protective equipment, such as goggles or face shields during procedures, was notably lower at 35%, suggesting a gap in the comprehensive application of PPE guidelines [6]. The underuse of comprehensive PPE could be due to discomfort, limited availability, or insufficient training on its importance [6,9]. Our results align with a study conducted in Ghana in 2017, which found that the practice of recapping needles is discouraged due to the risk of needle-stick injuries, indicating a need for enhanced education and vigilance in handling sharps [32].

Associations between basic characteristics of the study participants and KAP levels of infection control measures

This study found that good knowledge is significantly associated with being in clinical years of medical school (third, fourth, fifth, and internship). These findings are consistent with the study conducted among medical students at King Faisal University. Further analysis indicated that good practice was higher among female students, consistent with a study conducted in China [33]. This discrepancy may indicate the more cautious approach of female HCWs to infection control [6]. Female health professionals have been observed to exhibit higher compliance with safety precautions, which could be attributed to a greater perceived vulnerability or a more rigorous approach to patient care protocols. However, this was inconsistent with a study conducted among HCWs in Al-Qassim [12].

On the other hand, males have better knowledge of SICPs. This finding is similar to a study conducted in dental clinics in Jeddah in 2019 [18]. However, other studies report no significant differences [3,8,9,34]. The association between attending an infection control training program and good practice emphasizes the value of comprehensive training in improving SICP adherence [6,12,20]. This finding is corroborated by the findings of this study, which show a significant correlation between such training and good knowledge and practice scores.

In summary, many studies report that various factors influence compliance with infection control guidelines. More knowledge and positive attitudes toward standard infection precautions may be needed to ensure compliance [2]. Other factors, such as ongoing education sessions, adequate training, availability of resources, culture, workload, effective communication, and role models, play important roles [5,6,9]. Addressing these factors to promote adherence to SICP guidelines and enhance patient safety is crucial.

Study limitations

The study has some limitations. As with most questionnaire-based studies, there is potential bias from self-reported data and limitations of the KAP measurement tool. There could be a reporting bias that influenced the responses received from students to under or overestimate their KAP. Moreover, the questionnaire used by the study was relatively long, which could lead to participant fatigue or affect the quality of their responses. Another limitation could be that this is a single institution study, and it might be challenging to generalize it to the entire Kingdom of Saudi Arabia.

## Conclusions

In this study, medical students and interns at King Saud University showed unsatisfactory KAP toward SICPs. This drawback indicates a gap between the recommended guidelines and their implementation in healthcare settings, highlighting the necessity for integrating infection control education throughout the medical curriculum. Therefore, continuous education and practical training are imperative to equip medical students with the necessary skills and attitudes for effective infection control.
